# Age as a Mortality Predictor in ECPR Patients

**DOI:** 10.3390/medicina60091444

**Published:** 2024-09-04

**Authors:** Radim Spacek, Vojtech Weiss, Petra Kavalkova, Otakar Jiravsky, Jan Barcak, Jan Belohlavek

**Affiliations:** 1 Department of Cardiology, Hospital AGEL-Trinec Podlesi a.s., 739 61 Trinec, Czech Republic; 2 Third Faculty of Medicine, Charles University, 128 08 Prague, Czech Republic; 3 Department of Anesthesiology, Resuscitation and Intensive Medicine, First Faculty of Medicine, Charles University in Prague and General University Hospital, 12 808 Prague, Czech Republic; 4 2nd Department of Medicine—Department of Cardiovascular Medicine, First Faculty of Medicine, Charles University in Prague and General University Hospital, 12 808 Prague, Czech Republic; 5 Faculty of Medicine, Masaryk University, 601 77 Brno, Czech Republic

**Keywords:** ECMO, ECPR, extracorporeal, cardiopulmonary, resuscitation, age, outcomes

## Abstract

Extracorporeal cardiopulmonary resuscitation (ECPR) is an advanced technique using extracorporeal membrane oxygenation (ECMO) to support patients with refractory cardiac arrest. Age significantly influences ECPR outcomes, with younger patients generally experiencing better survival and neurological outcomes due to many aspects. This review explores the impact of age on ECPR effectiveness, emphasizing the need to consider age alongside other clinical factors in patient selection. Survival rates differ notably between in-hospital (IHCA) and out-of-hospital cardiac arrest (OHCA), highlighting the importance of rapid intervention. The potential of artificial intelligence to develop predictive models for ECPR outcomes is discussed, aiming to improve decision-making. Ethical considerations around age-based treatment decisions are also addressed. This review advocates for a balanced approach to ECPR, integrating clinical and ethical perspectives to optimize patient outcomes across all age groups.

## 1. Introduction to ECPR in Modern Cardiac Care

Extracorporeal cardiopulmonary resuscitation (ECPR) represents a vital advancement in the management of refractory cardiac arrest. This technique employs extracorporeal membrane oxygenation (ECMO) to provide hemodynamic and respiratory support to patients who do not respond to standard resuscitation efforts.

However, the deployment of ECPR is also associated with substantial resource utilization. Implementing ECPR requires not only sophisticated equipment and facilities but also a highly trained multidisciplinary team including physicians, nurses, perfusionists, and support staff [1]. The financial implications are also considerable, encompassing the costs of the technology itself, ongoing maintenance, and the training necessary to ensure that staff are proficient in its use. Therefore, effectivity of the care in terms of returning patients to their previous life is of utmost importance.

The impact of age on ECPR outcomes is notably significant, necessitating a deeper understanding of its role as a mortality predictor [2,3]. Older patients, who are generally characterized by a higher prevalence of comorbidities, frailty, and diminished physiological reserves, present unique challenges and considerations in the use of ECPR. Addressing how age affects survival rates and recovery quality is essential for refining patient selection and improving treatment protocols, ensuring that the substantial resources allocated to ECPR are used effectively and ethically.

This review aims to explore the multifaceted relationship between age and ECPR outcomes by analyzing current research, clinical insights, and ethical discussions. It aims to provide a comprehensive perspective on age as a critical factor in determining the success of ECPR.

## 2. Mortality Predictors in ECPR

Mortality predictors in ECPR patients encompass a range of clinical and demographic factors. Patients presenting with shockable rhythms (like ventricular fibrillation or pulseless ventricular tachycardia) often have better outcomes compared to those with non-shockable rhythms (such as asystole or pulseless electrical activity). Additionally, shorter durations between cardiac arrest and the start of ECPR are associated with improved survival and neurological outcomes, underscoring the importance of timely intervention [4,5]. Understanding mortality predictors in extracorporeal cardiopulmonary resuscitation (ECPR) is crucial for enhancing patient outcomes and refining clinical decision-making processes.

## 3. The Specific Role of Age

Age stands out as a particularly influential factor in most acute cardiac care conditions. As individuals age, their physiological capacity to recover from severe cardiac events diminishes, often complicating the recovery process and affecting the overall effectiveness of ECPR. Older patients typically have a higher burden of chronic health conditions, such as cardiovascular disease, diabetes, and kidney dysfunction, which can adversely impact their response to the strenuous demands of protracted CPR and complications of ECPR.

Studies consistently show that younger patients tend to have better survival rates and neurological outcomes following ECPR, highlighting the role of age as a crucial consideration in patient selection [2,3,6]. This age-related difference in outcomes is not only due to the physical challenges but also to the complexity of managing comorbidities in older patients. Therefore, understanding how age interacts with other clinical factors should provide essential insights into optimizing ECPR utilization and improving prognosis for patients of all ages.

## 4. ECPR Outcomes across Different Groups

The survival of patients in treating refractory cardiac arrest using ECPR varies significantly depending on many variables. As stated before—the time from cardiac arrest to ECMO start is one of the most important predictors. It is therefore unsurprising that there are significant differences in survival depending on where the arrest occurs—whether in the ECPR-capable hospital (in-hospital cardiac arrest—“IHCA”) or outside of it (out-of-hospital cardiac arrest—“OHCA”). While survival of patients suffering OHCA is usually lower compared to IHCA, the exact numbers vary, depending on local resources, ECPR team expertise, and study methodology. When the current literature is put together, IHCA survival usually falls down to somewhere between 20 and 60%, while survival in OHCA patients is significantly lower in a range of 15–35%, with very rare outliers [7,8,9,10,11].

## 5. Survival and Recovery across Age Groups

The optimal age cut-off values for ECPR have not been established, and the heterogeneity of studies is significant. The currently largest randomized trial for ECPR, the Prague OHCA study [9], had age of 18–65 as an inclusion criterion, which was violated in many cases due to difficulties to estimate real age in an OHCA setting. Other studies used 70, and even 75 years of age, as an upper cut-off value—all randomized ECPR trials are summarized in Table 1. Based on the available data, the Extracorporeal Life Support Organization recommends using the age of 70 as an upper limit [12].

Non-randomized data in this field are very heterogenous. Reaching firm conclusions is thus very precarious. We summarize these studies in Table 2. The largest of these is a study by Inoue et al. from Japan. They looked retrospectively on their cohort of 1644 ECPR OHCA patients. The median age in this study was 60 years, and ECPR was performed in patients up to 93 years. Complications were frequent, observed in 32% of patients. Factors associated with higher mortality were age, sex, initial shockable rhythm, and the location of cardiac arrest. Age was an independent predictor of mortality even after adjusting for different rhythms.

A study from the Republic of Korea, by Kim et al., was specifically focusing on the recommended age upper limit for ECPR. They reviewed 318 patient records and divided them into two groups, below or above 66 years of age. The older group was found to have double the mortality. It must be stated, however, that the rate of ECMO-related complications did not differ between the groups and that the survival with good neurological outcome was still 25% in the older patients.

Interesting results can be observed in another Japanese study by Miyamoto et al. Using the national registry, they looked at 875 OHCA patients undergoing ECPR, dividing them into three groups according to age: 18–59; 60–74; 75 and more. Survival rates were poor—15%, 8.9%, and 1.7%, respectively. Even though there was a substantial number of patients in the oldest group—115—CPR by a bystander was performed in only 29% of these cases, compared to 51% in the youngest group. Also, acute coronary syndrome was listed less frequently as a cause of cardiac arrest in the oldest group, resulting in coronary angiography being performed much more sparingly. These factors could have certainly led at least partially to the increased mortality in the older group.

Hsi-Yu Yu et al. looked at a relationship of low-flow time (the time from the start of CPR to the start of VA ECMO), age, and mortality. They divided patients into three groups: 18–65; 66–75; 76 and more. Low-flow times did not differ between the groups, and interestingly, favorable neurological outcome was the same for groups 1 and 2, and statistically non-significantly lower in the oldest group.

When the data are summed up, the use of ECPR might be seen as futile in certain older individuals where the likelihood of poor outcome is high, especially in people older than 75. However, denying ECPR solely based on age could prevent some patients from accessing a potentially life-saving intervention. This is especially critical in cases where older individuals maintain good physiological health and could realistically achieve a favorable outcome. Thus, the challenge lies in balancing the ethical imperative to do no harm with the potential to deny beneficial treatment based on chronological age.

## 6. Balancing Clinical Decision-Making and Ethical Imperatives

In clinical practice, the decision to provide ECPR must be fast and carefully consider both the potential benefits and the risks of harm. Optimally, it involves evaluating the patient’s overall health, their potential for recovery, and the likelihood of achieving meaningful quality of life post -intervention. In many cases, however, especially in OHCA patients, this is almost impossible. It is not a rarity that the medical team performing CPR in OHCA has no or very little information about the actual patient, forcing them to depend on other non-patient-centered data like whether the arrest was witnessed, whether there was a bystander CPR, etc. This is one of the reasons why it is imperative to daily neuroprognosticate and also evaluate the overall condition of patients to sustain critical illness post ECPR to eventually withdraw life-sustaining therapies in whom the prognosis is futile.

Moreover, it is very important to take into account the clinical setting of the cardiac arrest. A 75-year-old patient with OHCA far from an ECMO-capable hospital, with expected low-flow time significantly exceeding 60 min, has dismal prognosis. On the other hand, an 80-year-old patient might suffer from refractory arrest during a planned PCI due to technical challenges—CPR is started immediately, ECMO is readily available, leading to absent no-flow and short low-flow time, and when the ECMO is started, the coronary complication can usually be fixed right away. The prognosis of such elderly patient can be acceptable.

## 7. Research Gaps and Future Directions

Current research has significantly advanced our understanding of ECPR, but there remain substantial gaps that need addressing, particularly in the development of predictive tools. Age is a critical factor in determining outcomes of ECPR, yet it should not be considered in isolation. There is a pressing need to integrate age with other variables into a comprehensive, multivariable, but also simple to use, prediction score. Such scores could more accurately predict mortality and help in making nuanced decisions about who might benefit most from ECPR. There are many different scoring systems for various intensive care situations, but we are still missing a useful one for ECPR.

The development of sophisticated multivariable prediction models for ECPR outcomes could be greatly enhanced by leveraging artificial intelligence (AI) and machine learning techniques. AI has the potential to analyze large datasets from ECPR studies to identify patterns and predictors of survival that might be missed by traditional statistical methods. Machine learning algorithms can handle complex interactions between multiple variables, including subtle nuances that define different age groups’ responses to ECPR. The SCARS model [16], which can be used for conventional non-ECPR OHCA patients, is one of the already developed systems while using machine learning.

The application of AI in developing predictive scores highlights the need for more comprehensive and high-quality data on ECPR. Future research should focus on expanding and standardizing data collection in ECPR interventions, ensuring that datasets are robust and inclusive enough to train AI models effectively. Additionally, there is a need to conduct prospective studies to validate these AI-generated prediction scores, ensuring that they are clinically reliable and applicable across different healthcare settings.

Moreover, ethical considerations around the use of AI in clinical decision-making must also be addressed. Ensuring transparency in how these models are developed and applied, and maintaining a focus on patient-centered outcomes, will be crucial as these technologies become more integrated into clinical practice.

## 8. Conclusions

This review has comprehensively examined the critical role of age in the outcomes of extracorporeal cardiopulmonary resuscitation (ECPR), providing an understanding of how it influences survival and recovery across different patient groups. Age has been shown to be a significant predictor of both mortality and recovery quality, with younger patients generally experiencing better outcomes due to fewer comorbidities and greater physiological resilience.

Our exploration of ECPR outcomes across age groups revealed substantial differences in survival rates, highlighting the importance of timely and effective ECPR application, particularly for younger patients who demonstrate higher survival rates. However, the discussion also acknowledged that older patients, under certain conditions, could still benefit from ECPR, especially those who have maintained a high level of physical health and have minimal comorbid conditions.

The development of multivariable prediction scores that include age as a factor seems to be the way forward to help decision-making in this time-intensive field. The potential of artificial intelligence and machine learning to enhance these models is substantial. Future directions for research should include not only the refinement of predictive models but also a focus on expanding the evidence base for ECPR, particularly through the integration of large datasets that can train and test AI-driven tools. Such advancements are expected to improve decision-making processes, tailoring them more closely to individual patient profiles.

In conclusion, while the implementation of ECPR presents numerous challenges, particularly in the management of older patients, it remains a vital procedure with the potential for significant benefit events in the well-selected elderly. As healthcare continues to advance, especially with the incorporation of new technologies and data-driven approaches, the strategies for applying ECPR are expected to become increasingly sophisticated, enabling more personalized and effective care.

## Figures and Tables

**Table 1:** Age inclusion criteria for patients in all the randomized ECPR trials up to this date (CPC—cerebral performance category).

Name of the Study	Number of Patients (Intervention/Control)	Mean Age of Randomized	Age—Inclusion Criteria	30 Day CPC 1–2 in the Intervention Group
Prague OHCA [9]	124/132	58	18–65	31%
ARREST [10]	15/15	59	18–75	21%
INCEPTION [11]	70/64	54	18–70	20%

**Table 2:** Non-randomized studies on ECPR with a focus on age as a mortality predictor.

Study	Number of Patients	Inclusion Years	Main Conclusions
Miyamoto et al. [2]	875	2014–2017	Survival of 1.7% in older than 75; Less bystander CPR in the oldest group (29%, versus 51% in the youngest)
Hsi-Yu Yu et al. [3]	482	2006–2016	Favorable neurological outcomes not statistically different between age groups—27%, 24%, and 18%, respectively, for groups 18–65, 66–75, 76 and more
Inoue et al. [13]	1644	2013–2018	Patients up to 93 years old were cannulated; Favorable neurological outcome in 14%
Kim et al. [6]	318	2006–2018	Patients 66 years and older had double mortality compared to the younger group
Stub et al. [8]	26	Pre2013	Median age 52; Survival with good neurological outcome 54%
Goto et al. [14]	144	2005–2013	Worse survival in patients older than 70, OR 0.14 (95% CI, 0.03–0.8)
Chahine et al. [15]	391	2015–2023	30% higher mortality per every 10 years of age; Neurologically favorable survival 23% in the oldest group (70–79)
